# In vivo imaging of chronic active lesions in multiple sclerosis

**DOI:** 10.1177/1352458520958589

**Published:** 2020-09-23

**Authors:** Alberto Calvi, Lukas Haider, Ferran Prados, Carmen Tur, Declan Chard, Frederik Barkhof

**Affiliations:** Queen Square Multiple Sclerosis Centre, Department of Neuroinflammation, UCL Queen Square Institute of Neurology, University College London, London, UK/Unità di neurologia, Associazione Centro ‘Dino Ferrari’, IRCCS Fondazione Ca’ Granda Ospedale Maggiore Policlinico, University of Milan, Milan, Italy; Queen Square Multiple Sclerosis Centre, Department of Neuroinflammation, UCL Queen Square Institute of Neurology, University College London, London, UK/Department of Biomedical Imaging and Image-Guided Therapy, Medical University of Vienna, Vienna, Austria; Queen Square Multiple Sclerosis Centre, Department of Neuroinflammation, UCL Queen Square Institute of Neurology, University College London, London, UK/Centre for Medical Image Computing, Department of Medical Physics and Biomedical Engineering, University College London, London, UK/e-Health Centre, Universitat Oberta de Catalunya, Barcelona, Spain; Queen Square Multiple Sclerosis Centre, Department of Neuroinflammation, UCL Queen Square Institute of Neurology, University College London, London, UK/Neurology Department, Luton and Dunstable University Hospital, Luton, UK; Queen Square Multiple Sclerosis Centre, Department of Neuroinflammation, UCL Queen Square Institute of Neurology, University College London, London, UK/National Institute for Health Research (NIHR) University College London Hospitals (UCLH) Biomedical Research Centre, UK; Queen Square Multiple Sclerosis Centre, Department of Neuroinflammation, UCL Queen Square Institute of Neurology, University College London, London, UK/Centre for Medical Image Computing, Department of Medical Physics and Biomedical Engineering, University College London, London, UK/Radiology & Nuclear Medicine, VU University Medical Centre, Amsterdam, The Netherlands

**Keywords:** Multiple sclerosis, chronic active lesions, imaging, magnetic resonance imaging, positron emission tomography

## Abstract

New clinical activity in multiple sclerosis (MS) is often accompanied by acute inflammation which subsides. However, there is growing evidence that a substantial proportion of lesions remain active well beyond the acute phase. Chronic active lesions are most frequently found in progressive MS and are characterised by a border of inflammation associated with iron-enriched cells, leading to ongoing tissue injury. Identifying imaging markers for chronic active lesions in vivo are thus a major research goal. We reviewed the literature on imaging of chronic active lesion in MS, focussing on ‘slowly expanding lesions’ (SELs), detected by volumetric longitudinal magnetic resonance imaging (MRI) and ‘rim-positive’ lesions, identified by susceptibility iron-sensitive MRI. Both SELs and rim-positive lesions have been found to be prognostically relevant to future disability. Little is known about the co-occurrence of rims around SELs and their inter-relationship with other emerging techniques such as dynamic contrast enhancement (DCE) and positron emission tomography (PET).

## Introduction

White matter lesions (WMLs) are the hallmark of multiple sclerosis (MS) and the formation of new lesions underlies clinical relapses, however, in the long-term disability appears to be more closely linked with brain and spinal cord atrophy than WML accrual. It has become apparent that after their first formation some WMLs exhibit chronic activity and that the presence of chronic lesion activity is associated with clinical progression. Given this, they could represent a feature of MS lesions that could be worthwhile assessing using magnetic resonance imaging (MRI) in clinical trials and practice.

Histopathologically, the main features of WMLs are demyelination, axonal transection (in the acute phase) and degeneration (chronically), with accompanying gliosis. Recognising that lesions go through phases and run different course in the longer term, there have been several classification systems over the years, based on the presence of myelin degradation products and the distribution of inflammatory cells (macrophages/microglia, B-cells and T-cells).^1–4^ Four main MS lesion subtypes have been identified: early active; chronic active (also referred to as mixed active/inactive, slowly expanding or ‘smouldering’); inactive; and remyelinated (sometimes referred to as ‘shadow’) lesions. Early in the life of a new MS lesion, as found on autopsy in acute MS,^5^ the blood brain barrier (BBB) is breached, and extensive inflammatory activity and early myelin degradation products within macrophages are seen.^1–4^ Subsequently, some lesions remyelinate,^6^ other becomes inactive and some remain demyelinated.^7^

**Figure 1.**
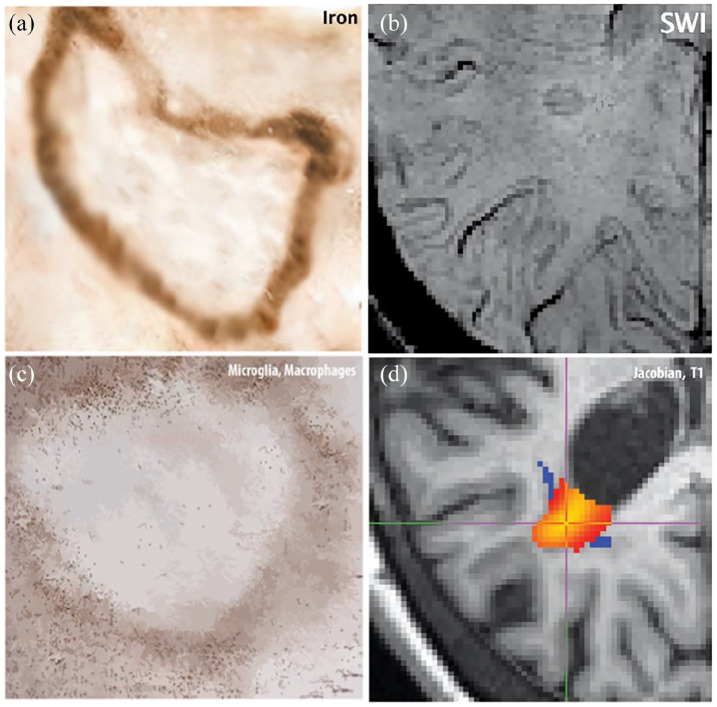
Chronic active lesion pathology-imaging features: Panel (a) shows a cartoon of the iron deposition at the edge of a chronic active lesion and panel (b) shows an example of a hypointense rim on a susceptibility-weighted scan probably reflecting iron. Panel (c) shows a cartoon of activated microglia/macrophages in the periphery of a chronic active lesion and we assume that this inflammatory activity is responsible for low expansion of SEL lesion visible in (d).

Chronic active lesions are a subset of MS lesions with inactive demyelinated centres, which additionally maintain or develop continuous myelin breakdown at the edge, with expansion towards the surrounding white matter (Figure 1). They represent one of the most common lesion types seen in histopathological studies of people with primary progressive (PP) and secondary progressive (SP) MS and may constitute up to ~30% of the total WML burden.^8,9^ A higher proportion of chronic active lesions has been associated with higher overall lesion load and a shorter time to reach disability milestones.^8^ The accumulation of chronic active lesions with persistent inflammation provides a plausible missing link between WMLs and longer term neurodegeneration.

MRI is the principal method used to support a diagnosis of MS and to assess treatment efficacy in early phase MS clinical trials. It is highly sensitive in detecting WMLs on conventional T2-weighted (T2), proton density (PD), fluid attenuated inversion recovery (FLAIR) and T1-weighted (T1) sequences, but not specific for histological subtypes/stages.^10^ New T2 lesions correspond with early active lesions and early on may show transient contrast enhancement on T1 (due to gadolinium leakage, typically lasting 2–6 weeks).^11^ Persistent black holes (PBHs), defined by T1 hypointensity lasting for at least 6–12 months, are associated pathologically with greater reductions in myelin and axonal density,^10,12^ when compared with WMLs that are not T1 hypointense.

Despite correlation with relapses,^13^ the overall WML load only partially correlates with disability in long term^14–16^ (*r* ranging from 0.13 to 0.67), and while variable degrees of remyelination may help to explain this,^17^ neurodegeneration seemingly occurring independently of lesions (as depicted on MRI as brain and spinal cord atrophy)^18,19^ appears to be the major driver of long-term disability in MS.

Several quantitative MRI techniques have been developed that can assess tissue microstructure, such as magnetization transfer ratio (MTR) and diffusion-weighted imaging (DWI), as shown by imaging-pathological correlations.^20–23^ PBH,^24,25^ lesion MTR^26–28^ and DWI-derived measures^29^ all correlate with disability and brain atrophy but the relationship of these markers to chronic lesion activity remains unknown.

Here, we review developments in the MRI assessment of chronic active lesions, focussing on ‘slowly expanding lesions’ (SELs) and rim-positive lesions as seen on susceptibility MRI and exploring future potential applications of dynamic contrast enhancement (DCE) and PET imaging. An effective imaging biomarker of chronic lesion activity would offer a more comprehensive assessment of ongoing lesion activity, potentially allow us to identify people with MS at risk of developing progressive MS and provide us with tool with which to assess an additional aspect of treatment effectiveness.

## Methods

We sought to identify all published studies on the radiological aspects of chronic active lesions in MS adults.

PubMED and EMBASE were separately searched using the following terms: ‘chronic active lesion’ or ‘expanding/smouldering lesion’ combined with ‘multiple sclerosis’. To this first screening, we added either ‘imaging’, inclusive of the magnetic resonance imaging and positron emission tomography (PET). All studies available in English and published before 1 April 2020 were included.

From 66 articles initially identified, after excluding papers focussing on other neurological conditions (e.g. neuromyelitis optica spectrum disorder) or paediatric MS, case reports, animal model or biomolecular markers studies, we identified 15 studies. A further 15 studies satisfying our inclusion criteria were identified by cross-referencing from the 15 studies already found.

## SELs

Few studies have been published on this topic to date. They are based on the premise that ongoing tissue injury at lesion edges is associated with lesion expansion and that this can be detected by volumetric assessment of WMLs on longitudinal MRI.

Subtraction MRI allows us to more readily assess regional tissue changes between scans, when compared with simply visually comparing separate scans and so helps us to identify new or enlarging WMLs.^30^ However, this method does not allow to accurately track chronic lesions as it requires visual assessment limited by interobserver variability. Automatic detection techniques, such as voxel-guided morphometry, have proven able to identify chronic enlarging and shrinking lesions, finding them to be correlated with local brain atrophy,^31^ reinforcing the view that specific WML types could make different contributions to MS disability progression.

More recent studies have used deformation-based techniques to assess tissue deformation (as a Jacobian map) at a sub-voxel level and have enabled SELs to be defined as WMLs with a constant and concentric volume increase.^32^ Constancy requires that expansion persists over time, as observed by progressive volume change over multiple time points. Concentricity is determined by a pattern of expansion with a preferential direction towards the external boundaries of the lesion. Overall, the sum of the two criteria provides a highly specific marker, avoiding inclusion of lesions with volume fluctuations. Results from pooled trial cohorts^33^ (*n* = 2388) indicate that a higher proportion of SELs is seen in PPMS compared with relapsing-remitting MS (RRMS), in line with the pathological findings. Gadolinium enhancement was higher in WMLs not classified as SEL (non-SEL), while SELs had lower T1 intensity at baseline with a larger signal decrease at follow-up, suggesting presence of myelin and axonal loss.^33^ In a subsequent study in PPMS (*n* = 732), SELs had the lowest T1 intensity values and accounted for a higher amount of T1 hypointense volume accumulation.^34^ Moreover, SEL volume could predict progression on a composite disability measure.^34^

## Susceptibility imaging to track chronic active lesions: rim-positive lesions

Susceptibility-weighted MRI techniques allow the identification of paramagnetic and diamagnetic substances such as iron-related proteins and myelin. The main techniques include simple gradient-echo T2*-weighted sequences (T2*) and more complex techniques incorporating phase and magnitude information such as susceptibility-weighted imaging (SWI) and quantitative susceptibility mapping (QSM).

Lesions surrounded by a rim of hypointense signal, also referred to as ring-like or rim-positive lesions, were first described on visual inspection of T2* and SWI.^35,36^ A dark rim, seen in a subgroup of WMLs, corresponds to a peripheral phase shift at 7 T,^36,37^ and this has been translated to 3 T MRI.^38^ Several histopathological-imaging studies have confirmed an association between rim-positivity and chronic active lesions.^37,39,40^

Cross-sectional susceptibility MRI studies in MS have reported that between 10% and 20% of lesions are rim-positive.^36,41^ Longitudinal studies describe at least one rim-positive lesion in the majority of patients, in both relapsing and progressive MS.^42,43^ Rim-positivity is associated to higher overall mean WML volume and progressive accumulation of T1-hypointensity.^44^ A recent study on T2* and phase MRI stratified MS patients according to number of rim-positive lesions^45^ and found that having ⩾4 rim-positive lesions was associated with reaching motor and cognitive disability milestones at a younger age and more rapid brain atrophy. At an individual lesion level, rim-positive lesions are also more likely to grow compared with lesions that do not have a rim.^46^

R2* (transverse relaxivity – inverse of T2*) is a susceptibility-based quantitative measure that correlates with both myelin density and tissue iron contents.^47^ Increased R2* co-localises with rim-positivity and has been linked with peripheral iron deposition in chronic active lesions.^37^ An alternative quantitative approach is QSM, a post-processing technique removing phase artefacts, which can be computed from all susceptibility-based sequences. The advantage of QSM is that the phase information distinguishes myelin from iron: studies of chronic active lesions have found a positive QSM value with iron deposition and a QSM close to zero associated with a reduction in myelin content.^48^ WMLs can be staged according to QSM features: phase values are similar to normal appearing white matter (NAWM) in new active lesions, progressively increase in the early chronic stage and finally return to normal values in the late chronic stage.^49^

## DCE

DCE studies, utilising repeated post-contrast T1-weighted serial images rapidly acquired during and after gadolinium injection, have shown that early lesions demonstrate a centrifugal-DCE (nodular) pattern around a central vein, consistent with initial opening of BBB. Conversely, older and larger lesions are marked by a centripetal-DCE (shell) pattern, reflecting capillary recruitment at the lesion edge and outward expansion.^50,51^

By combining DCE and susceptibility-imaging studies, chronic non-enhancing MS lesions have been found to be more often associated with susceptibility rims.^40^ However, ‘transient’ rims have been detected in the initial stages of lesion formation, reflecting first infiltration of macrophages/microglia carrying paramagnetic substances.^43^ Both centrifugal and centripetal-enhancing patterns are associated with rim-positivity and its persistence.^45,44^ Persistence of rim (seen in up to 20% of the lesions)^44^ might represent a further marker of chronic active lesion inflammation.

In quantitative susceptibility analyses, nodular-enhancing lesions have QSM values close to zero, while shell-enhancing and chronic lesions show increasing QSM values related to lesion age.^52^ Early- and intermediate-age lesions are associated with lower R2* values when compared with chronic lesions, and shell-enhancing type has the largest R2* relative decrease. Since concurrent R2* changes and DCE abnormalities are not associated with QSM alterations, they probably reflect demyelination, while in chronic lesions both measures become abnormal, indicative of iron accumulation.

## PET

Using radiolabelled ligands binding specific targets, PET imaging can detect chronic MS lesions with ongoing inflammatory activity. First and second-generation PET radioligands are able to bind to the translocator protein (TSPO) expressed by microglia/macrophages, 18 kDa TSPO and lesser to astrocytes in diverse neurological conditions.^53^ In MS, histopathological studies have shown that TSPO uptake is a marker for chronic active lesions and have demonstrated an association with rim-positivity on susceptibility MRI.^54^

A recent study combining MRI and PET scans showed that rim-positive lesions on QSM showed a higher TSPO uptake as compared to rimless lesions.^54^ QSM rim-positivity and PET uptake co-localised to high pro-inflammatory activity and iron deposition within chronic active lesions.

## Discussion

There are now several MRI techniques that can be used to identify chronically active lesions in MS. Some assess dynamic features, such as lesion expansion, and other characteristics of lesions that are or have been active, for example, susceptibility rims.

SEL detection represents a promising marker for chronic active lesions, as it can be undertaken using routinely acquired MRI scans. However, the SEL detection relies on a heuristically determined lesion expansion threshold rate, based on Jacobian values, and there may still be scope for this to be optimised. The second parameter to select SEL, concentricity, relies on the hypothesis of homogeneous peripheral expansion. However, in pathological descriptions, portions of the lesion border might expand without involving the whole perimeter.^55^ In addition, the use of various scanner resolutions might affect SEL detection and the number of scanning time points is also relevant. Despite the limitations of this technique, the correlation with disability progression suggests that chronic brain inflammation may largely be driven by the accumulation of SELs.

Over longer periods of time (5–10 years), it is possible that lesions could follow different trajectories, rather than all passing through the same stages in a monotonic manner. This may depend on inflammatory and atrophic pathological components, which could be reflected in lesion morphology features such as their shape or size. Volumetric MRI analysis can not only detect lesions expansion but also detect shrinkage, and recent studies have reported an overall tendency for chronic lesions to shrink,^56^ suggestive of chronic lesion degeneration, and the presence of shrinking lesions in early MS,^57^ consistent with the resolution of inflammation and associated tissue oedema. More recently, WML atrophy has been observed particularly in periventricular brain regions^58^ and has been found to correlate with confirmed disability progression^59,60^ and higher risk of conversion to SPMS.^61^

Susceptibility-imaging MRI studies combined with post-mortem analysis indicated a linkage between rim-positivity and presence of iron-enriched cells^44–46^ (mainly ferritin within oligodendrocytes and macrophages/microglia). The percentages of rim-positive lesions identified in those studies (~10%–20%) are slightly lower than those for chronic active lesions in pathological studies (~30%). Susceptibility changes are also observed in NAWM, suggesting that variability of iron deposition and myelin density is not exclusively a feature of focal WMLs.^39^ Higher numbers of rim-positive lesions are associated to clinical relapses in RRMS,^62^ and their association with clinical disability suggests that rim-positive lesions might also represent a risk factor for MS patients developing clinically progressive disease.^45^ Interestingly, rim-positivity was found more often in expanding lesions, which might link the two MRI modalities (Figure 1), both detecting two different features of chronic inflammatory activity.^46^

While T2* imaging has proven sensitive in the qualitative detection of rim-positive lesions, other measures can provide valuable additional information. R2* is affected by both myelin (diamagnetic) and iron (paramagnetic) concentrations, and in isolation, this make it difficult to discern the evolution these features in chronic active lesions, however, combining this with QSM provides additional value, since reduced myelin and iron accumulation induce opposite effects on phase-shifts, and it is also substantially affected by relatively small amounts of iron.^63^

DCE studies have shown that initial demyelination in active lesions proceeds from a central vein, visualised as nodular-enhancing pattern^40^ and that this is followed by opening of the peripheral capillaries corresponding to shell-enhancing pattern, characterised by low R2* (consistent with reduced myelin content) and increased lesion dimensions in intermediate stages.

Persistence of rims, confirmed by QSM findings, might be linked to accumulation of paramagnetic substances, such as iron, eventually leading to prolonged pro-inflammatory environment and failure of tissue protection.^62^ Active inflammatory cells at chronic active lesions border have been detected using PET imaging and the effects of this persisting inflammatory activity are reflected by volumetric MRI expansion, visualised as SELs. The end-stage phase of WMLs might involve a plateau of expansion, with the accumulation of atrophic components, favouring tissue collapse and ultimately volume shrinkage.

In conclusion, multiple different imaging approaches confirm the presence of chronic active lesions in MS, and together they highlight that a substantial proportion of lesions show features of chronic activity (lesion volumes changes, the presence of susceptibility rims or both). Correlations between volumetric and susceptibility features, supported by findings from DCE and PET studies, suggest that robust MRI markers for chronic active lesion are becoming available. These may provide a novel, and clinically relevant, perspective on treatment response and so deserve being considered for use in future clinical trials.
